# The efficacy of acupuncture on endometrial receptivity in infertile women: an overview of systematic review and meta-analysis

**DOI:** 10.3389/fmed.2025.1609519

**Published:** 2025-08-28

**Authors:** Huaying Fan, Jie Zhou, Chenjian Tang, Huan Liu

**Affiliations:** ^1^Hospital of Chengdu University of Traditional Chinese Medicine, Chengdu, China; ^2^The First Affiliated Hospital of Traditional Chinese Medicine of Chengdu Medical College, Chengdu, China; ^3^Xindu Hospital of Traditional Chinese Medicine, Chengdu, China; ^4^TCM Preventative Treatment Research Center of Chengdu Medical College, Chengdu, China; ^5^Nanbu County People’s Hospital, Nanchong, China

**Keywords:** acupuncture, endometrial receptivity, infertility, overview, systematic review

## Abstract

**Background and objective:**

Endometrial receptivity (ER) enhancement is crucial in managing infertility. Although systematic reviews (SRs) have investigated acupuncture’s potential to improve ER in infertile women, the evidence remains fragmented due to insufficient quality assessment. This overview aimed to rigorously evaluate the reporting quality, methodological rigor, risk of bias, and evidence confidence of existing SRs, while synthesizing clinical evidence on acupuncture’s efficacy and safety for ER enhancement in infertility.

**Search strategy:**

Seven databases (PubMed, Embase, Web of Science, Cochrane Library, CNKI, VIP, Wanfang) were systematically searched from inception to March 1, 2025, using combined subject headings and free-text terms (“acupuncture therapy,” “endometrial receptivity,” “infertility,” “systematic review”).

**Inclusion criteria:**

SRs investigating acupuncture’s therapeutic effects on ER in infertile women were eligible.

**Data extraction and analysis:**

Two independent reviewers performed study selection and data extraction. Methodological quality, reporting completeness, bias risk, and evidence certainty were evaluated using validated tools: AMSTAR 2, ROBIS, PRISMA-A, and GRADE.

**Results:**

From 524 screened records, 10 SRs (published 2019–2023, encompassing 7–25 RCTs each) were included. Methodological quality assessed by AMSTAR 2 showed that all 10 SRs exhibited critically low quality. Reporting quality assessed by PRISMA-A showed that overall completeness >70%, but deficiencies in protocol registration (50%) and funding disclosure (10%). Risk of bias assessed by ROBIS showed that only one SR had low risk of bias. As to the evidence confidence, among the 55 evaluated outcomes, 92.72% (51/55) were low/very low quality (2 high, 2 moderate, 24 low, 27 very low). Descriptive analyses suggested that combining acupuncture with other treatments (medications, Chinese herbal medicine, and IVF-ET) may improve pregnancy and ovulation rates, with a high to moderate quality of evidence.

**Conclusion:**

Current evidence supporting acupuncture for ER enhancement is predominantly low quality, limited by critical methodological weaknesses and heterogeneity. While combination therapies show preliminary promise, definitive conclusions require high-quality RCTs with standardized outcome measures.

**Systematic review registration:**

This study was registered in PROSPERO (CRD42024497881).

## 1 Introduction

Infertility, defined as the inability to achieve clinical pregnancy after 12 months of unprotected intercourse, is a major global public health challenge (1). Affecting approximately 15% of couples worldwide with a rising incidence (2–4), infertility significantly impacts women’s physical and psychological health and creates societal burdens, including social stigma, marital instability, and financial strain (5–8). While assisted reproductive technology (ART) offers treatment, clinical pregnancy rates (CPR) remain suboptimal, largely due to embryonic developmental abnormalities and poor endometrial receptivity (PER). Notably, PER contributes to roughly two-thirds of implantation failures (9, 10), highlighting its clinical importance.

Endometrial receptivity (ER) refers to the transient state of the uterus that allows blastocyst implantation. It is typically assessed using three main parameters: endometrial thickness (EMT), morphology, and vascular perfusion. Establishing ER involves complex interactions involving hormones, cytokines, growth factors, and uterine blood flow (11). Since PER is a key cause of female infertility, recurrent miscarriage, and poor ART outcomes, improving ER is crucial for addressing infertility and enhancing ART success rates (12, 13).

Current strategies to enhance ER include: (1) hormonal interventions (oral/vaginal estrogen) to stimulate endometrial growth; (2) medications like low-dose aspirin, low-molecular-weight heparin, or sildenafil citrate to improve blood flow; and (3) mechanical interventions (intrauterine perfusion/physical stimulation) to increase ER sensitivity. Despite these approaches, outcomes are often inadequate. Existing treatments frequently show limited effectiveness, poor patient compliance (especially with long-term heparin), and significant side effects, from gastrointestinal issues to thromboembolic risks (12, 13). This clear therapeutic gap underscores the urgent need for novel, more effective, and safer treatments.

Acupuncture is increasingly used in infertility care. Survey indicate 75% of UK complementary medicine clinics offer acupuncture for fertility support (14), with proposed mechanisms including regulating ovarian function, restoring hormonal balance, and reducing stress (15–17). Studies suggest acupuncture may improve ER through multiple pathways: enhancing endometrial structure, increasing uterine blood flow, regulating progesterone signaling, and modulating key molecular markers (e.g., integrin αvβ3, LIF, VEGF, HOXA10) essential for implantation (15, 18–22). However, systematic reviews (SRs) on acupuncture’s efficacy for ER improvement report conflicting results, likely due to methodological differences and inconsistent reporting in primary studies.

The value of SR evidence depends on rigorous methods and transparent reporting (23). Although numerous SRs examine acupuncture for ER optimization, comprehensive quality assessments are still scarce. Therefore, we conducted an overview of SRs to systematically evaluate their reporting quality, risk of bias, methodological quality, and confidence levels. We further investigated the efficacy and safety of acupuncture for improving ER in women with infertility. Unlike prior reviews, this study is the first overview of the systematic reviews to rigorously evaluate ER-specific outcomes across infertility subtypes.

## 2 Methods

### 2.1 Protocol and registration

This study was registered on PROSPERO (CRD42024497881). As it involved the analysis of existing SRs, ethical approval was not required. This overview adhered strictly to the registered protocol. No deviations occurred; all planned analyses (quality appraisal, narrative synthesis) were executed.

### 2.2 Literature search

A systematic search was conducted across seven electronic databases from their inception to March 1, 2025: PubMed, Embase, Web of Science, Cochrane Library, China National Knowledge Infrastructure (CNKI), Chongqing VIP Chinese Science and Technology Periodical Database (VIP), and Wanfang. The search strategy utilized standardized subject headings and free-text terms encompassing four core concepts: “acupuncture therapy,” “endometrial receptivity,” “infertility,” and “systematic review.” Detailed search strings for the four international databases (PubMed, Embase, Web of Science, Cochrane Library) are provided in Supplementary material 1. Search strategies for Chinese databases were tailored to their specific query structures.

### 2.3 Inclusion and exclusion criteria

#### 2.3.1 Study design

SRs adhering to PRISMA guidelines were eligible if they synthesized evidence from two or more randomized controlled trials (RCTs) investigating acupuncture interventions for improving ER in women with infertility. SRs were excluded if they were a network meta-analyses, a protocol of meta-analysis, or full text was not available to be reviewed. Network meta-analyses were excluded to avoid confounding from indirect comparisons; protocols were excluded as they lack synthesized data.

#### 2.3.2 Participants

Infertile women with PER were included, provided uterine factors were confirmed by transvaginal sonography (TVS) without structural abnormalities. Exclusion criteria comprised congenital uterine anomalies, intrauterine adhesions, or active pelvic inflammatory disease.

#### 2.3.3 Intervention

Interventions in the experimental group mainly included acupuncture or acupuncture plus other treatments such as medications, Chinese herbal medicine (CHM), *in vitro* fertilization-embryo transfer (IVF-ET), embryo transfer (ET), and frozen embryo transfer (FET). The acupuncture types included manual acupuncture, electrical acupuncture (EA), warm acupuncture, moxibustion, acupoint catgut implantation, abdominal acupuncture, transcutaneous electrical acupuncture stimulation (TEAS), plum-blossom needle, auricular point sticking, auricular acupuncture, and acupoint injection.

#### 2.3.4 Comparators

The comparators included medicine, placebo, sham acupuncture (SA), lifestyle, physiotherapy, routine care, IVF-ET, ET, FET, CHM, and no treatment.

#### 2.3.5 Outcomes

Fertility outcomes, ER outcomes and safety outcomes were included. Specifically, fertility outcomes included pregnancy rate (PR), CPR, live birth rate (LBR), biochemical pregnancy rate (BPR), ovulation rate, embryo transfer number/rate, and embryo implantation number/rate; ER outcomes included endometrial pattern (EMP), EMT, endometrial pulse index (PI), resistive index (RI), and peak systolic velocity/end-diastolic blood velocity (S/D).

### 2.4 Study selection

A comprehensive literature search was performed following the predetermined search strategy. All retrieved records were imported into EndNote X9 (Clarivate Analytics, United States) for systematic data management. After automated deduplication, two reviewers (LH, ZJ) independently screened records in two stages: (1) titles/abstracts to exclude clear irrelevancies; (2) full texts of potentially eligible articles. Strict exclusion criteria were applied at each stage, with reasons documented per PRISMA guidelines. To ensure methodological rigor, the reviewers performed cross-verification of their screening decisions upon completion. Any discrepancies in study selection were resolved through panel discussion, with unresolved disagreements arbitrated by a senior researcher (FHY).

### 2.5 Data extraction

A dual extraction protocol was implemented using a standardized extraction template. Two investigators (LH and TCJ) independently extracted the following data elements from included systematic reviews: (1) basic characteristics: authorship, publication year, country of origin, funding sources; (2) methodological components: search databases utilized, number of RCTs/participants, diagnostic criteria, intervention protocols, outcome measures, risk of bias assessment tools; (3) synthesis findings: primary conclusions and safety profiles. All extractions underwent cross-validation, with discordant entries resolved through structured reconciliation meetings. Persistent discrepancies were adjudicated by a senior investigator (FHY) to ensure consensus.

### 2.6 Methods

#### 2.6.1 Assessment of methodological quality

A Measurement Tool to Evaluate Systematic Review 2 (AMSTAR 2) was applied to assess the methodological quality of the included SRs. AMSTAR 2 is a critical appraisal tool for evaluating the quality of SRs, allowing for a comprehensive evaluation of SRs in terms of study selection, protocol design, data extraction, data analysis, and discussion. AMSTAR2 consists of 16 entries with seven key entries (2, 4, 7, 9, 11, 13, 15) and nine non-key entries. Among the 16 entries, five of the entries (2.4.7.8.9) are evaluated with “yes,” “partially yes” and “no,” and the remaining entries were evaluated with “yes” and “no.” SRs was categorized as high, medium, low and very low based on the conformity of the entries. The evaluation criteria are as follows: (1) rated as “high”: no or one non-critical weakness; (2) rated as “medium”: more than one non-critical weakness; (3) rated as “low”: one critical weakness with or without non-critical weaknesses; (4) rated as “very low”: more than one critical weakness with or without non-critical weaknesses (24). Two assessors (ZJ and TCJ) independently conducted evaluations. All discordant ratings underwent blinded re-evaluation followed by consensus discussion, with final arbitration by a senior investigator (FHY) when required.

#### 2.6.2 Risk of bias assessment

The risk of bias of included SRs were assessed using Risk of Bias in Systematic Review tool (ROBIS) which is completed in three phases, with the phase−1 focusing on assessing relevance which is optional, the phase-2 focusing on identifying concerns during the review process, and the phase-3 focusing on judging the risk of bias (25). The phase-2 comprises four domains, including study eligibility criteria, identification and selection of studies, data collection and study appraisal, and synthesis and findings. Phase-3 evaluates the overall risk of bias in the interpretation of review results. The risk of bias was rated as “low,” “high,” or “unclear.” Two independent investigators (LH and ZJ) assessed the ROBIS and reviewed each other’s findings once completed. Any differences in scores that were not resolved through discussion were assessed by a senior investigator (FHY).

#### 2.6.3 Assessment of reporting quality

Reporting quality was assessed using Preferred Reporting Items for Systematic Review and Meta-analyses for Acupuncture (PRISMA-A) checklist. PRISMA-A is a tool for improving the reporting quality of SRs related to acupuncture, which contains 27 items with each item is answered with “yes,” “no,” or “partly yes” (26). Two independent investigators (LH and TCJ) assessed the PRISMA-A separately with a cross-check progress in the end, and a senior investigator (FHY) was involved if disagreements about assessment results could not be solved by discussion.

#### 2.6.4 Assessment of evidence

Evidence confidence of included SRs was assessed by the Grades of Recommendation, Assessment, Development, and Evaluation (GRADE). To ensure consistency, the GRADE rating was reapplied to all outcomes included in the systematic review. GRADE encompasses five aspects: risk of bias, inconsistency, indirection, imprecision, and other considerations (publication bias, large effect, plausible confounding, and dose–response gradient).

Evidence quality is categorized into four levels: High (very confident that the true effect is close to the estimated value; well-designed RCTs typically start as high), Moderate (moderately confident that the true value is likely close to the estimate but there is a possibility of difference), Low (limited confidence that the true value may differ substantially from the estimate), and Very low (little to no confidence that the true value is likely substantially different from the estimate).

The key to determining the evidence quality level is the “downgrading” or “upgrading” of a predefined starting point (high for RCTs, low for observational studies). Downgrading factors include: (1) Study limitations/Risk of bias (flaws in study design or execution); (2) Inconsistency (large heterogeneity in results between studies that cannot be reasonably explained); (3) Indirectness (differences in the PICO elements—Population, Intervention, Comparison, Outcome—between the studies and the target question); (4) Imprecision (small sample size or low event number leading to overly wide confidence intervals); (5) Publication bias (indications that studies with negative results were not published). Upgrading factors (primarily applicable to observational studies starting from low) include: (1) Large magnitude of effect (e.g., RR ≥ 2 or <0.5 that cannot be readily explained by confounding); (2) Dose–response relationship; (3) All plausible confounding factors would diminish the observed effect. The assessment process was performed for each critical outcome, starting from the initial level, and comprehensively considering the downgrading and upgrading factors to arrive at the final grade.

This assessment was conducted independently by two researchers (ZJ and TCJ) using the GRADEpro GDT platform,^1^ followed by cross-verification. Any discrepancies in ratings were resolved through group discussion or arbitration by a senior researcher (FHY).

### 2.7 Heterogeneity analysis

Clinical and methodological heterogeneity across SRs (e.g., population differences, acupuncture protocols) precluded meta-analysis. We followed SWiM guidelines for narrative synthesis, categorizing results by outcome and intervention type.

## 3 Results

### 3.1 Literature search

**Figure 1:**
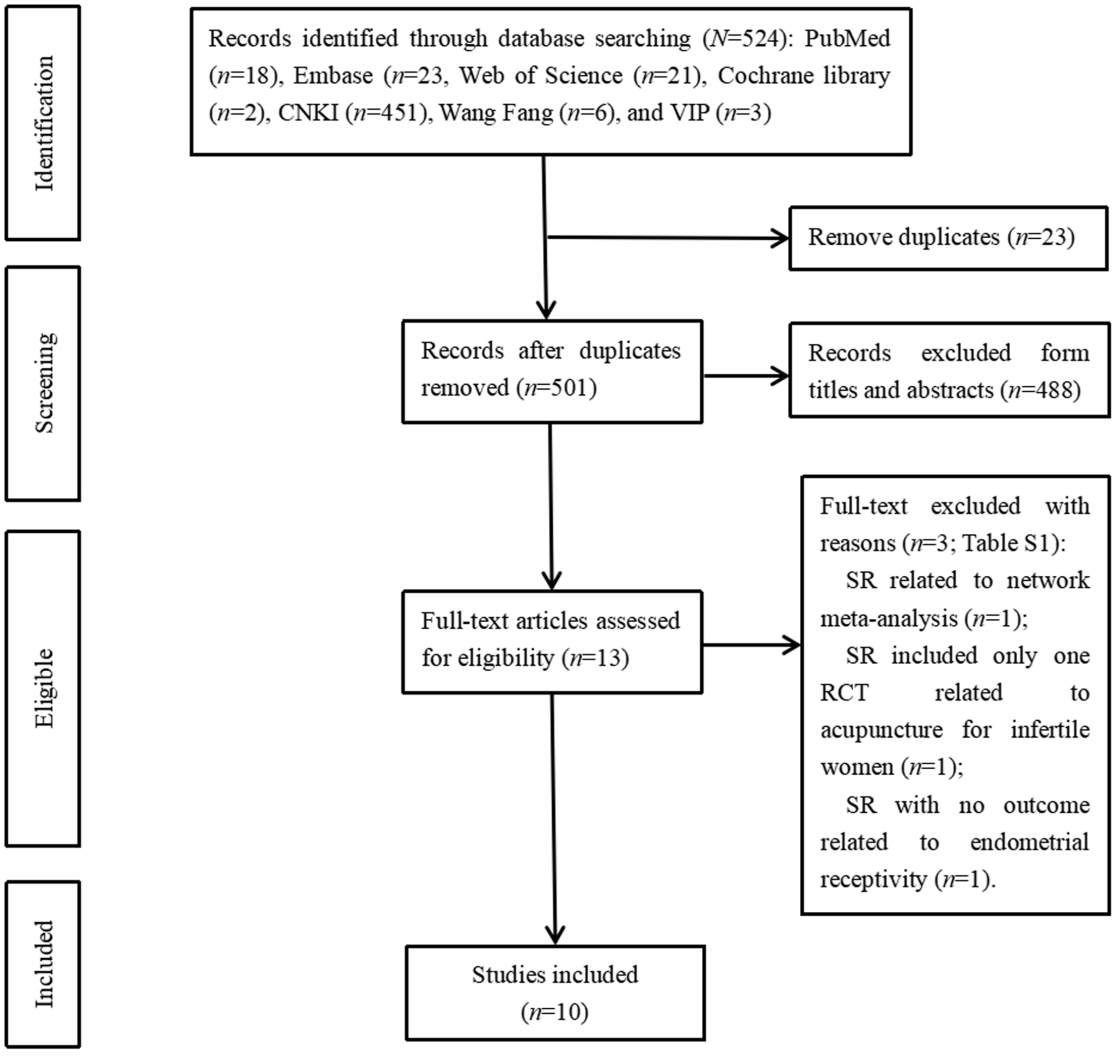
The inclusion and exclusion of systematic reviews studying acupuncture on endometrial receptivity in infeltile women. CNKI, China National Knowledge Infrastmcture; VIP, Chongqing VIP Chinese Science and Technology Periodical Database.

Following searches across seven databases, 524 records were identified: PubMed (*n* = 18), Embase (*n* = 23), Web of Science (*n* = 21), Cochrane Library (*n* = 2), CNKI (*n* = 451), WanFang (*n* = 6), and VIP (*n* = 3). After removing 23 duplicates, 501 records underwent screening. Title/abstract assessment yielded 13 potentially eligible articles. Full-text review excluded three articles: one lacking ER-related outcomes (27), one being a network meta-analysis (28), and one containing only a single acupuncture RCT for infertility within its SR. Ten articles met all inclusion criteria (29–38). The literature selection process is detailed in Figure 1.

### 3.2 Characteristic of the included SRs

Characteristics of the 10 included systematic reviews (2019–2023) are summarized in Table 1. Two SRs were published in Chinese and eight in English. The included SRs searched six to 12 databases and included seven to 25 RCTs with the participants varied from 516 to 3,041. All 10 SRs performed meta-analysis, eight of 10 SRs performed subgroup analysis, and seven SRs performed sensitivity analysis. The treatment groups included acupuncture alone, acupuncture plus medicine/CHM, acupuncture plus medicine and CHM, acupuncture plus IVF-ET, ET, or FET, acupuncture plus IVF-ET and CHM, acupuncture combined with IVF-ET, and intrauterine shortwave therapy (IST). The acupuncture methods included manual acupuncture, warm acupuncture, TEAS, plum-blossom needle treatment, EA, cupping, auricular point sticking, auricular acupuncture, acupoint injection, acupoint catgut embedding, and moxibustion. The control groups included the following interventions, either singly or in combination: medicine, SA, blank control, routine care, physiotherapy, press bean combined with electromagnetic wave lamp, CHM, IVF-ET, ET, FET, and shallow acupuncture. SA included sham acupuncture, mock transcutaneous electrical acupuncture stimulation (MTEAS), sham electroacupuncture, placebo acupuncture, placebo auricular acupuncture, and placebo moxibustion. The medicine included Diane-35, metformin, placebo, human chorionic gonadotrophin (HCG), bromocriptine, progesterone, ethinylestradioland cyproterone acetate tablets, hormone replacement therapy, levofloxacin, dydrogesterone, letrozole, clomiphene, estradiol valerate tablets, controlled ovarian hyperstimulation, aspirin and gentamicin, dexamethasone, chymotrypsin, sodium chlorideL. The outcome measures mainly referred to fertility outcomes (PR, CPR, LBR, embryo implantation rate, fertilization rate, ovulation rate, number of oocytes retrieved, maximum follicle diameter, high-quality embryo rate, antral follicle count, and number of embryo transfers), ER outcomes (endometrial vascular index, S/D, RI, PI, endometrial flow index, EMP, and EMT), endocrine outcomes (FSH, LH, P, E2, and AMH), and other outcomes (efficacy rate, Gn duration, Gn dose, and cycle cancelation rate). All 10 SRs were assessed using the Cochrane bias risk assessment tool.

**Table 1:** The characteristics of the included systematic reviews.

Study	Language	Included databases	N/n	Intervention	Control	Outcomes	Methodological quality assessment tool	Meta-analysis	Subgroup analysis	Sensitivity analysis	Safety
Zhong 2019 (29)	English	PubMed, Embase, CENTRAL, Web of Science, KCI, Clinical Trials. gov. con, SinoMed, VIP BIOSIS Previews, J-STAGE, CNKI, and WFDP	*N* = 13 *n* = 3,041	acu; acu + Meds	Meds; SA; routine care; press bean+ electromagnetic wave lamp	B + C + D + F + G + H + J + O + S	Cochrane bias risk assessment tool	Yes	Yes	Yes	Yes
Li 2021 (30)	English	PubMed, Embase, CNKI, VIP, Web of Science, WFDP, SinoMed, and Cochrane Library	*N* = 7 *n* = 756	acu; acu + Meds; acu + TDP + Meds; acu + FET	SA + Meds; Meds; FET; SA/black	F + G + H + J + Z + a	Cochrane bias risk assessment tool	Yes	Yes	No	Yes
Liu 2019 (31)	English	MEDLINE, Embase, Cochrane Central Register of Controlled Trials, CNKI, VIP, and WFDP	*N* = 22 *n* = 2,591	acu; acu + Meds+CHM; acu + Meds/CHM	Meds; Meds+placebo	G + H + L + P + R	Cochrane bias risk assessment tool	Yes	Yes	Yes	Yes
Liu 2020 (32)	English	PubMed, Cochrane Library, VIP, Embase, WFDP, and CNKI	*N* = 10 *n* = 715	acu; acu + Meds; acu + Meds+CHM	Meds; Meds +CHM	C + D + H + L + R + S	Cochrane bias risk assessment tool	Yes	No	Yes	Yes
Yahui 2022 (33)	Chinese	PubMed, Embase, WFDP, VIP, SinoMed, CNKI, and Web of Science	*N* = 13 *n* = 915	acu + IVF-ET; acu + IVF-ET + IST/CHM	IVF-ET; IVF-ET + CHM	G + H	Cochrane bias risk assessment tool	Yes	Yes	Yes	No
Zheng 2022 (34)	English	PubMed, Embase, Cochrane Library, Web of Science, VIP, SinoMed, CNKI, and WFDP	*N* = 14 *n* = 1,564	acu + IVF-ET/ET/FET/Meds	IVF-ET; FET; Meds; IVF-ET/FET + SA; shallow acu + FET	A + B + C + D + E + F + G + H + I	Cochrane Collaboration’s tool	Yes	Yes	Yes	No
Zhu 2022 (35)	English	Cochrane Library, PubMed, Embase, SinoMed, CNKI, and VIP	*N* = 14 *n* = 1,130	acu + FET	FET + no adjuvant treatment/SA	F + G + H + I + a	Cochrane risk of bias assessment tool	Yes	Yes	Yes	Yes
Liyuan 2023 (36)	Chinese	PubMed, Embase, CNKI, WFDP, Web of Science, VIP, Sinomed, and Cochrane Library	*N* = 25 *n* = 1920	acu + CHM; acu + CHM + Meds	Meds	G + H + L	Cochrane risk of bias assessment tool	Yes	Yes	No	No
Mo 2023 (37)	English	PubMed, Embase, CNKI, WFDP, Web of Science, Sinomed, and Cochrane Library	*N* = 21 *n* = 1841	acu + CHM; acu + CHM + Meds	Meds	G + H + L + N + P + Q + S + V	Cochrane risk of bias assessment tool	Yes	Yes	No	Yes
Wang 2023 (38)	English	MEDLINE (via PubMed), CNKI, Embase, Sinomed, VIP, WFDP, and Allied and Complementary Medicine Database	*N* = 7 *n* = 516	acu + Meds/IVF	Meds; IVF	G + H + J + K + M + O + P + R + S + T + U + W + X + Y	Cochrane risk of bias assessment tool	Yes	No	Yes	Yes

KCI, Korean Citation Index; J-STAGE, Japan Science and Technology Information Aggregator, Electronic; CNKI, China National Knowledge Infrastructure; VIP, Chinese Science and Technology Periodical Database; SinoMed, China Biomedical Literature Service System; WFDP, Wan Fang database; N, number of included randomized controlled trials; n, number of participants; acu, acupuncture (including one of manual acupuncture, warm acupuncture, transcutaneous electrical acupuncture stimulation, plum-blossom needle treatment, electroacupuncture, cupping, auricular point sticking, auricular acupuncture, acupoint injection, acupoint catgut embedding, and moxibustion); Meds, medicine (including one of or some combinations of diane-35, metformin, placebo orally, human chorionic gonadotrophin, bromocriptine, progesterone, ethinylestradioland cyproterone acetate tablets, hormone replacement therapy, levofloxacin, dydrogesterone, letrozole, clomiphene, estradiol valerate tablets, controlled ovarian hyperstimulation, aspirin and fallopian tube injection of gentamicin, dexamethasone, chymotrypsin, sodium chlorideL); CHM, Chinese herbal medicine; IVF-ET, in vitro fertilization and embryo transfer; IST, intrauterine shortwave therapy; FET, frozen embryo transfer; ET, embryo transfer; SA, sham acupuncture (including mock transcutaneous electrical acupuncture stimulation, sham electroacupuncture, placebo acupuncture, placebo auricular acupuncture, placebo moxibustion acupuncture); A, endometrial vascular index; B, peak systolic velocity/end-diastolic blood velocity; C, resistive index; D, endometrial pulse index; E, endometrial flow index; F, endometrium pattern; G, endometrial thickness; H, pregnancy rate; I, live birth rate; J, embryo implantation rate; K, fertilization rate; L, ovulation rate; M, number of oocytes retrieved; N, maximum follicle diameter; O, high-quality embryo rate; P, follicle stimulating hormone; Q, progesterone; R, luteinizing hormone; S, estradiol; T, anti-mullerian hormone; U, antral follicle count; V, efficacy rate; W, duration of Gn; X, Dose of Gn; Y, Cycle cancelation rate; Z, number of embryo transfers;a, biochemical pregnancy rate.

### 3.3 Quality of the included SRs

#### 3.3.1 Quality of the methodology

**Figure 2:**
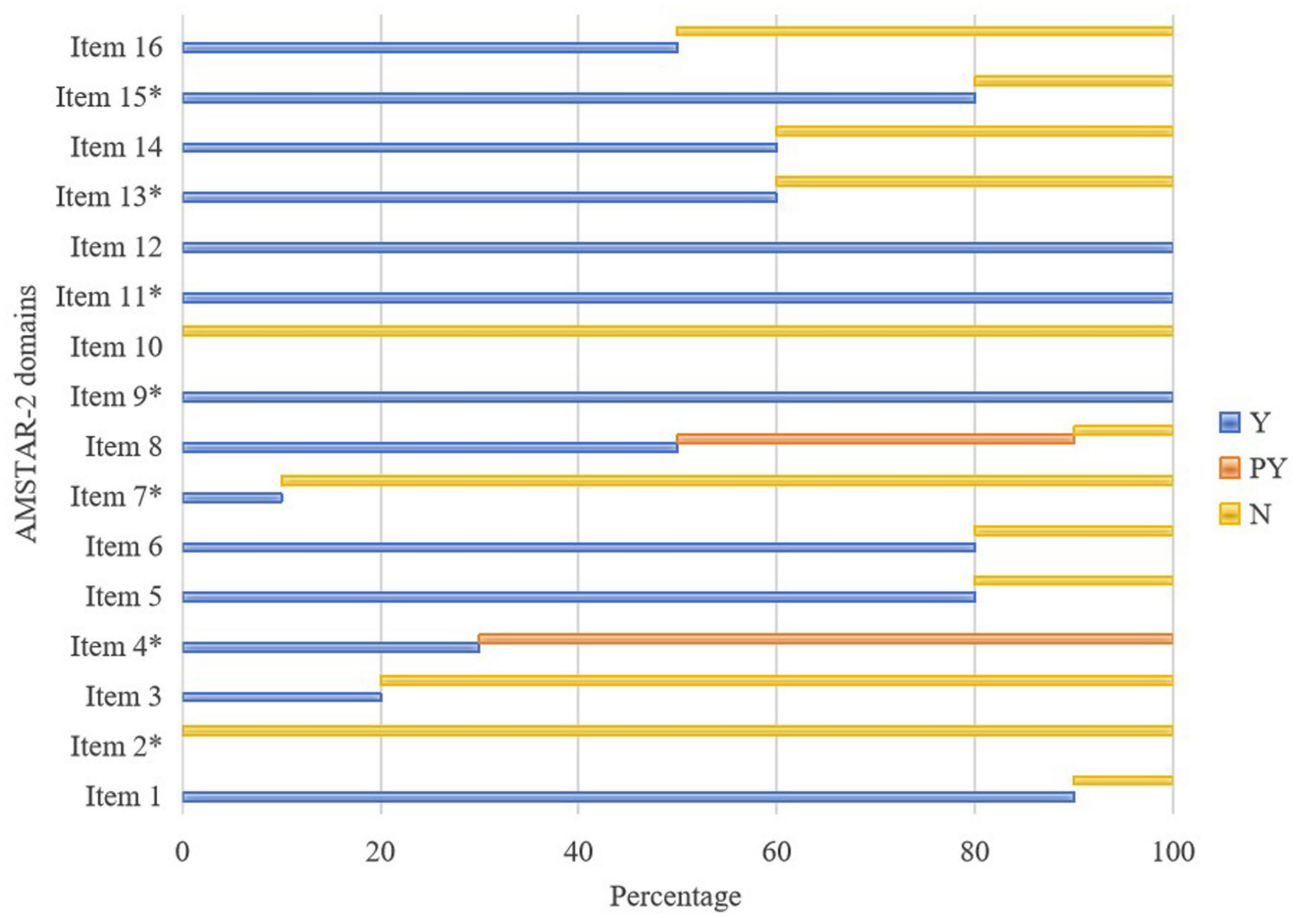
Graphical presentation ofAMSTAR-2. Y, yes; PY, partial yes; N, no; AMSTAR2, A Measurement Tool to Evaluate Systematic Review 2.

All SRs failed critical AMSTAR-2 domains: 100% lacked *a priori* protocols (Item 2) and funding disclosures (Item 10); 90% omitted excluded study lists (Item 7). Table 2 and Figure 2 present the results.

**Table 2:** Methodological assessments of included systematic reviews by AMSTAR 2.

Item	Study									
	Zhong 2019	Li 2021	Liu 2019	Liu 2020	Zhang 2022	Zheng 2022	Zhu 2022	Jiao 2023	Mo 2023	Wang 2023
Item 1	Y	Y	Y	N	Y	Y	Y	Y	Y	N
Item 2*	N	N	N	N	N	N	N	N	N	N
Item 3	N	N	Y	N	N	Y	N	N	N	N
Item 4*	PY	PY	PY	PY	PY	Y	Y	PY	Y	PY
Item 5	Y	Y	Y	N	Y	Y	N	Y	Y	Y
Item 6	Y	N	Y	Y	Y	Y	Y	N	Y	Y
Item 7*	N	N	N	N	N	N	N	N	N	Y
Item 8	Y	PY	PY	Y	PY	Y	N	Y	PY	Y
Item 9*	Y	Y	Y	Y	Y	Y	Y	Y	Y	Y
Item 10	N	N	N	N	N	N	N	N	N	N
Item 11*	Y	Y	Y	Y	Y	Y	Y	Y	Y	Y
Item 12	Y	Y	Y	Y	Y	Y	Y	Y	Y	Y
Item 13*	Y	Y	Y	N	N	Y	Y	N	Y	N
Item 14	Y	Y	N	N	Y	Y	Y	N	Y	Y
Item 15*	Y	N	Y	Y	Y	Y	Y	Y	Y	N
Item 16	Y	N	N	Y	N	N	Y	N	Y	Y
Ranking of quality	Critically low	Critically low	Critically low	Critically low	Critically low	Critically low	Critically low	Critically low	Critically low	Critically low

Y, yes; N, no; PY, partial yes. *, the key items of the AMSTAR 2; AMSTAR, A Measurement Tool to Assess Systematic Reviews. Item 1: Did the research questions and inclusion criteria for the review include the components of PICO? Item 2: Did the report of the review contain an explicit statement that the review methods were established prior to the conduct of the review and did the report justify any significant deviations from the protocol? Item 3: Did the review authors explain their selection of the study designs for inclusion in the review? Item 4: Did the review authors use a comprehensive literature search strategy? Item 5: Did the review authors perform study selection in duplicate? Item 6: Did the review authors perform data extraction in duplicate? Item 7: Did the review authors provide a list of excluded studies and justify the exclusions? Item 8: Did the review authors describe the included studies in adequate detail? Item 9: Did the review authors use a satisfactory technique for assessing the risk of bias (RoB) in individual studies that were included in the review? Item 10: Did the review authors report on the sources of funding for the studies included in the review? Item 11: If meta-analysis was performed, did the review authors use appropriate methods for statistical combination of results? Item 12: If meta-analysis was performed, did the review authors assess the potential impact of RoB in individual studies on the results of the meta-analysis or other evidence synthesis? Item13: Did the review authors account for RoB in primary studies when interpreting/discussing the results of the review? Item14: Did the review authors provide a satisfactory explanation for, and discussion of, any heterogeneity observed in the results of the review? Item 15: If they performed quantitative synthesis did the review authors carry out an adequate investigation of publication bias (small study bias) and discuss its likely impact on the results of the review? Item 16: Did the review authors report any potential sources of conflict of interest, including any funding they received for conducting the review?

#### 3.3.2 Risk of bias

The results of ROBIS assessment are shown in Table 3. In phase-2, all SRs had a low risk of bias in Domain−1 and Domain-4 and six SRs were considered to have a low risk of bias in Domain-3; notably, ROBIS showed high bias in study selection (Domain 2). In phase-3, only one SR had an overall low risk of bias.

**Table 3:** Results of the ROBIS assessment.

Section/topic	Zhong 2019	Li 2021	Liu 2019	Liu 2020	Zhang 2022	Zheng 2022	Zhu 2022	Jiao 2023	Mo 2023	Wang 2023
Phase 2-domain 1:study eligibility criteria										
2.1.1	PY	PY	PY	PY	PY	PY	PY	PY	PY	PY
2.1.2	Y	Y	Y	Y	Y	Y	Y	Y	Y	Y
2.1.3	Y	Y	Y	Y	Y	Y	Y	Y	Y	Y
2.1.4	Y	Y	Y	Y	Y	Y	Y	Y	Y	Y
2.1.5	Y	Y	Y	Y	Y	Y	Y	Y	Y	Y
Risk	L	L	L	L	L	L	L	L	L	L
Phase 2-domain 2: identification and selection of studies										
2.2.1	PY	PY	PY	PY	Y	Y	Y	PY	PY	Y
2.2.2	N	N	N	N	N	Y	N	N	Y	N
2.2.3	Y	Y	Y	Y	Y	Y	Y	PY	PY	Y
2.2.4	Y	PY	PY	PY	PY	N	PY	N	Y	PY
2.2.5	Y	Y	Y	PY	Y	Y	PY	Y	Y	Y
Risk	H	H	H	H	H	H	H	H	L	H
Phase 2-domain 3: collection and study appraisal										
2.3.1	Y	PN	Y	Y	Y	Y	Y	PN	Y	Y
2.3.2	Y	Y	Y	Y	Y	N	Y	Y	Y	Y
2.3.3	Y	Y	Y	Y	Y	Y	Y	Y	Y	Y
2.3.4	Y	Y	Y	Y	Y	Y	Y	Y	Y	Y
2.3.5	Y	Y	Y	N	Y	N	Y	N	Y	Y
Risk	L	H	L	H	L	H	L	H	L	L
Phase 2-domain 4: synthesis and findings										
2.4.1	Y	Y	Y	Y	Y	Y	Y	Y	Y	Y
2.4.2	PY	PY	PY	PY	PY	PY	PY	PY	PY	PY
2.4.3	Y	Y	Y	Y	Y	Y	Y	Y	Y	Y
2.4.4	Y	Y	Y	Y	Y	Y	Y	Y	Y	Y
2.4.5	Y	Y	Y	Y	Y	Y	Y	Y	Y	Y
2.4.6	Y	Y	Y	Y	Y	Y	Y	Y	Y	Y
Risk	L	L	L	L	L	L	L	L	L	L
Phase 3 risk if bias in the review										
A	N	N	N	N	N	N	N	N	Y	N
B	Y	Y	Y	Y	Y	Y	Y	Y	Y	Y
C	Y	Y	Y	Y	Y	Y	Y	Y	Y	Y
Risk	H	H	H	H	H	H	H	H	L	H

Y, yes; PY, probably yes; PN, probably no; N, no; L, low risk; H, high risk.

#### 3.3.3 Reporting quality

**Figure 3:**
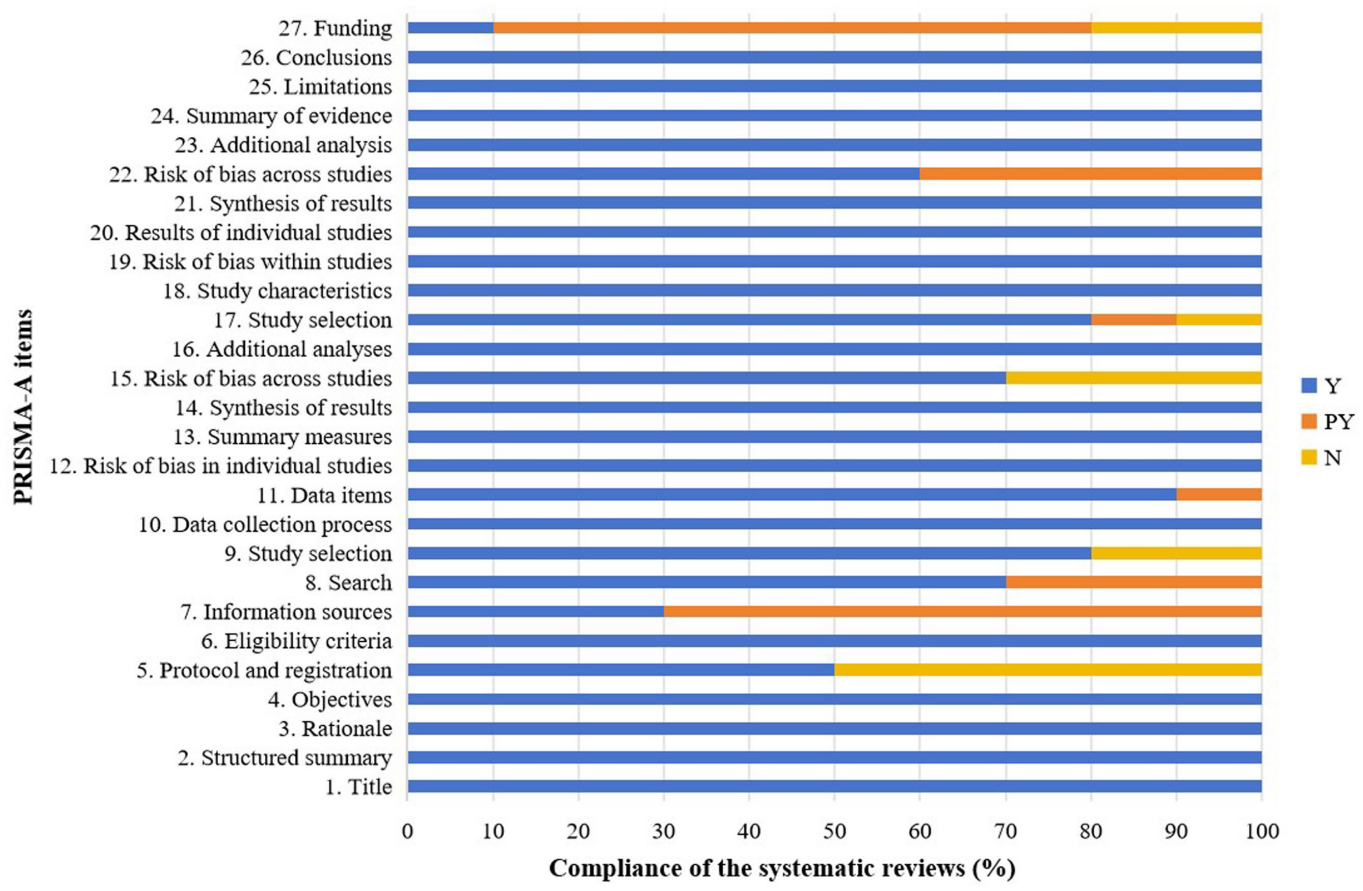
Graphical presentation of PRISMA-A. Y, yes; PY, partial yes; N, no; PRISMA-A, Preferred Reporting Items for Systematic Reviews and Meta-analyses for Acupuncture.

Reporting quality assessed by PRISMA-A showed that overall completeness >70%, suggesting that the reports were mostly complete (39), but deficiencies centered on protocol registration (Item5, 50% compliance) and funding disclosure (Item27, 10% compliance). A detailed assessment of PRISMA-A is shown in Table 4 and Figure 3.

**Table 4:** Compliance of included SRs with PRISMA-A checklist.

Items	Section/topic	Zhong 2019	Li 2021	Liu 2019	Liu 2020	Zhang 2022	Zheng 2022	Zhu 2022	Jiao 2023	Mo 2023	Wang 2023	Of yes (%) [n (%)]
Title	1. Title	Y	Y	Y	Y	Y	Y	Y	Y	Y	Y	10 (100%)
Abstract	2. Structured summary	Y	Y	Y	Y	Y	Y	Y	Y	Y	Y	10 (100%)
	3. Rationale	Y	Y	Y	Y	Y	Y	Y	Y	Y	Y	10 (100%)
	4. Objectives	Y	Y	Y	Y	Y	Y	Y	Y	Y	Y	10 (100%)
Method	5. Protocol and registration	Y	N	N	N	N	Y	Y	N	Y	Y	5 (50%)
	6. Eligibility criteria	Y	Y	Y	Y	Y	Y	Y	Y	Y	Y	10 (100%)
	7. Information sources	PY	PY	PY	PY	PY	Y	Y	PY	Y	PY	3 (30%)
	8. Search	Y	Y	Y	PY	Y	Y	Y	PY	PY	Y	7 (70%)
	9. Study selection	Y	Y	Y	N	Y	Y	N	Y	Y	Y	8 (80%)
	10. Data collection process	Y	Y	Y	Y	Y	Y	Y	Y	Y	Y	10 (100%)
	11. Data items	Y	PY	Y	Y	Y	Y	Y	Y	Y	Y	9 (90%)
	12. Risk of bias in individual studies	Y	Y	Y	Y	Y	Y	Y	Y	Y	Y	10 (100%)
	13. Summary measures	Y	Y	Y	Y	Y	Y	Y	Y	Y	Y	10 (100%)
	14. Synthesis of results	Y	Y	Y	Y	Y	Y	Y	Y	Y	Y	10 (100%)
	15. Risk of bias across studies	Y	Y	Y	N	N	Y	Y	Y	Y	N	7 (70%)
	16. Additional analyses	Y	Y	Y	Y	Y	Y	Y	Y	Y	Y	10 (100%)
Results	17. Study selection	Y	Y	PY	Y	Y	Y	Y	N	Y	Y	8 (80%)
	18. Study characteristics	Y	Y	Y	Y	Y	Y	Y	Y	Y	Y	10 (100%)
	19. Risk of bias within studies	Y	Y	Y	Y	Y	Y	Y	Y	Y	Y	10 (100%)
	20. Results of individual studies	Y	Y	Y	Y	Y	Y	Y	Y	Y	Y	10 (100%)
	21. Synthesis of results	Y	Y	Y	Y	Y	Y	Y	Y	Y	Y	10 (100%)
	22. Risk of bias across studies	Y	N	Y	N	Y	Y	Y	N	Y	N	6 (60%)
	23. Additional analysis	Y	Y	Y	Y	Y	Y	Y	Y	Y	Y	10 (100%)
	24. Summary of evidence	Y	Y	Y	Y	Y	Y	Y	Y	Y	Y	10 (100%)
	25. Limitations	Y	Y	Y	Y	Y	Y	Y	Y	Y	Y	10 (100%)
	26. Conclusions	Y	Y	Y	Y	Y	Y	Y	Y	Y	Y	10 (100%)
Funding	27. Funding	Y	N	PY	PY	N	PY	PY	PY	PY	PY	1 (10%)
	Of yes [n (%)]	26 (96.30)	22 (81.48)	23 (85.19)	20 (74.07)	23 (85.19)	26 (96.30)	25 (92.26)	21 (77.78)	25 (92.26)	23 (85.19)	

Y, yes; PY, probably yes; N, no.

#### 3.3.4 Confidence in study outcomes

Confidence in study outcomes were assessed by GRADE and the quality of outcomes varied from high quality to very low quality. Specifically, of these, two were of high quality, two were of moderate quality, 24 were of low quality, and 27 were of very low quality. Risk of bias, followed by publication bias, imprecision and inconsistency, was the most common reason for downgrading. The detailed results are presented in Table 5.

**Table 5:** Evidence quality of included studies.

Outcome	Study	Interventions vs. comparisons	Included RCTs (participants)	Effect (95% CI)	Quality assessment					
					Risk of bias	Inconsistency	Indirection	Imprecision	Other considerations	Quality of evidence
Fertility outcomes										
PR	Zhong 2019 (29)	acu vs. Meds/SA/routine care/press bean + electromagnetic wave lamp	6 (1,194/1242)	RR: 1.17 (1.07, 1.28)	−1①	0	0	0	−1②	Low
		acu + Meds vs. Meds	5 (248/225)	RR: 1.68 (1.30, 2.17)	−1①	0	0	0	−1②	Low
	Liu 2019 (31)	acu + Meds vs. Meds	5 (185/183)	RR: 1.86 (1.36, 2.54)	−1①	0	0	0	−1②	Low
		acu + CHM + Meds vs. Meds	4 (186/184)	RR: 1.52 (1.25, 1.86)	−1①	0	0	0	−1②	Low
		acu vs. Meds	3 (97/97)	RR: 2.63 (1.60, 4.32)	−1①	0	0	0	−1②④⑥	Low
		acu + CHM vs. Meds	8 (359/353)	RR: 1.99 (1.60, 2.46)	−1①	0	0	0	−1②	Low
	Liu 2020 (32)	acu vs. Meds	3 (90/88)	RR: 1.86 (0.93, 3.75)	−1①	0	0	−1③	−1②	Very low
		acu + Meds vs. Meds; acu + CHM + Meds vs. CHM + Meds	6 (216/210)	RR: 2.39 (1.71, 3.32)	−1①	0	0	0	0	Moderate
	Yahui 2022 (33)	acu + IVF-ET vs. IVF-ET; acu + IVF-ET + CHM vs. IVF-ET + CHM; acu + IVF-ET + IST vs. IVF-ET	11 (363/365)	RR: 2.41 (1.76, 3.30)	−1①	0	0	0	+1⑥	High
	Zheng 2022 (34)	acu + IVF-ET vs. IVF-ET; acu + IVF-ET vs. SA + IVF-ET; acu + FET vs. SA + FET; acu + FET vs. shallow acu + FET; acu + FET vs. FET; acu + Meds vs. Meds	14 (776/758)	RR: 1.97 (1.39, 2.79)	−1①	−1⑤	0	0	−1②	Very low
	Liyuan 2023 (36)	acu + CHM vs. Meds; acu + CHM + Meds vs. Meds	21 (820/818)	RR: 1.72 (1.51, 1.97)	−1①	0	0	0	−1②	Low
	Mo 2023 (37)	acu + CHM vs. Meds; acu + CHM + Meds vs. Meds	14 (620/612)	RR: 2.5 (1.96, 3.18)	−1①	0	0	0	+1⑥	High
CPR	Li 2021 (30)	acu + Meds/FET vs. Meds/FET; acu + Meds+TDP vs. SA + Meds; acu VS SA/blank	7 (303/376)	RR: 1.90 (1.51, 2.40)	−1①	0	0	0	−1④	Low
	Zhu 2022 (35)	acu + FET vs. FET + no adjuvant treatment/SA	14 (562/568)	RR: 1.54 (1.28, 1.85)	−1①	0	0	0	−1②	Low
	Wang 2023 (38)	acu + COH vs. COH	2 (58/54)	RR: 1.59 (0.80, 3.17)	−1①	0	0	−1④	−1④	Very low
BPR	Li 2021 (30)	acu + Meds/FET vs. Meds/FET; acu + Meds+TDP vs. SA + Meds; acu VS SA/blank	5 (207/280)	RR: 1.59 (1.27, 1.99)	−1①	0	0	0	−1④	Low
	Zhu 2022 (35)	acu + FET vs. FET	5 (177/177)	RR: 1.51 (1.21, 1.89)	−1①	0	0	0	−1④	Low
LBR	Zhong 2019 (29)	acu vs. SA/ routine care	2 (395/154)	RR: 1.47 (0.76, 2.83)	−1①	0	0	0	−1④	Low
	Zheng 2022 (34)	acu + FET vs. SA + FET; acu + FET vs. FET; acu + IVF-ET vs. SA + IVF-ET	3 (68/69)	OR: 2.96 (1.42, 6.16)	−1①	−1⑤	0	−1③	−1②	Very low
	Zhu 2022 (35)	acu + FET vs. FET + no adjuvant treatment/SA	4 (215/216)	RR:1.48 (0.90, 2.43)	−1①	−1⑤	0	0	0	Low
Ovulation rate	Liu 2019 (31)	acu + Meds vs. Meds; acu + CHM + Meds vs. Meds; acu vs. Meds; acu + CHM vs. Meds	15 (839/863)	RR: 1.29(1.21, 1.37)	①	0	0	0	−1②	Low
	Liu 2020 (32)	acu vs. Meds	3 (255/255)	RR: 1.57 (1.32, 1.86)	−1①	0	0	0	−1②	Low
	Liyuan 2023 (36)	acu + CHM vs. Meds; acu + CHM + Meds vs. Meds	11 (not mentioned)	RR: 1.04 (0.94, 1.16)	−1①	−1⑤	0	0	−1②	Very low
	Mo 2023 (37)	acu + CHM vs. Meds; acu + CHM + Meds vs. Meds	7 (307/297)	RR: 2.46 (1.72, 3.52)	−1①	0	0	0	0	Moderate
Embryo transfer	Zhong 2019 (29)	acu vs. Meds	2 (59/65)	RR:2.04 (1.13, 3.70)	−1①	0	0	③	-1④	Very low
	Li 2021 (30)	acu + Meds vs. Meds; acu + Meds+TDP vs. SA + Meds; acu VS SA/blank	4 (173/245)	MD: 0.02 (−0.08, 0.12)	-1①	0	0	0	-1④	Low
Embryo implantation	Li 2021 (30)	acu + Meds vs. Meds; acu + Meds+TDP vs. SA + Meds; acu vs. SA	5 (477/539)	RR: 1.89 (1.47, 2.45)	-1①	0	0	0	-1④	Low
	Wang 2023 (38)	acu + Meds vs. Meds	2(101/92)	RR: 2.13 (1.08, 4.21)	-1①	0	0	-1③	0	Low
Endometrial receptivity outcomes										
EMP	Zhong 2019 (29)	acu vs. Meds/SA/routine care/press bean+ electromagnetic wave lamp	1 (30/30)	RR: 1.50 (0.81, 2.79)	−1①	0	0	−2③	−1④	Very low
		acu + Meds vs. Meds	5 (248/244)	RR: 1.47 (1.26, 1.71)	−1①	0	0	0	−1②	Low
	Li 2021 (30)	acu + Meds/FET vs. Meds/FET	3 (169/170)	RR: 1.49 (0.70, 3.18)	−1①	−1⑤	0	0	−1④	Very low
	Zheng 2022 (34)	acu + IVF-ET vs. IVF-ET; acu + IVF-ET vs. SA + IVF-ET; acu + FET vs. SA + FET; acu + FET vs. FET; acu + Meds vs. Meds	9 (327/336)	OR: 2.48 (1.26, 4.90)	−1①	−1⑤	0	0	−1②	Very low
	Zhu 2022 (35)	acu + FET vs. FET + no adjuvant treatment/SA	7 (235/238)	RR: 1.41 (1.13, 1.75)	−1①	−1⑤	0	0	0	Low
EMT	Zhong 2019 (29)	acu vs. Meds/SA/routine care	3 (68/66)	SMD: 0.18 (−0.16, 0.52)	−1①	0	0	−1③	−1④	Very low
		acu + Meds vs. Meds	6 (268/264)	SMD: 0.52 (0.12, 0.93)	−1①	−1⑤	0	0	−1②	Very low
	Li 2021 (30)	acu + Meds/FET vs. Meds/FET; acu + Meds+TDP vs. SA + Meds; acu VS SA/blank	6 (278/345)	MD: 1.11 (0.59, 1.63)	−1①	−1⑤	0	0	−1④	Very low
	Liu 2019 (31)	acu + CHM + Meds vs. Meds; acu vs. Meds; acu + CHM vs. Meds	9 (310/308)	MD: 1.39 (0.51, 2.27)	−1①	−1⑤	0	0	−1②	Very low
	Yahui 2022 (33)	acu + IVF-ET vs. IVF-ET; acu + IVF-ET + CHM vs. IVF-ET + CHM; acu + IVF-ET + IST vs. IVF-ET	12 (389/379)	MD: 0.83 (0.22, 1.44)	−1①	−1⑤	0	0	−1②	Very low
	Zheng 2022 (34)	acu + IVF-ET vs. IVF-ET; acu + IVF-ET vs. SA + IVF-ET; acu + FET vs. SA + FET; acu + FET vs. shallow acu + FET; acu + FET vs. FET; acu + Meds vs. Meds	11 (531/529)	SMD: 0.48 (0.13, 0.83)	−1①	−1⑤	0	0	0	Low
	Zhu 2022 (35)	acu + FET vs. FET + no adjuvant treatment/SA	12 (415/421)	MD: 0.97 (0.43, 1.51)	−1①	−1⑤	0	0	0	Low
	Liyuan 2023 (36)	acu + CHM vs. Meds; acu + CHM + Meds vs. Meds	14 (not mentioned)	SMD: 1.10 (0.78, 1.42)	−1①	−1⑤	0	0	−1②	Very low
	Mo 2023 (37)	acu + CHM vs. Meds; acu + CHM + Meds vs. Meds	13 (381/384)	SMD: 1.75 (1.40, 2.10)	−1①	−1⑤	0	0	0	Low
	Wang 2023 (38)	acu + Meds vs. Meds	3 (110/115)	MD:0.54(0.13, 0.96)	−1①	0	−1⑦	−1③	0	Very low
RI	Zhong 2019 (29)	acu vs. press bean plus electromagnetic warm lamp	1 (30/30)	MD: −0.02 (−0.03, −0.01)	−1①	0	0	−1③	−1④	Very low
		acu + Meds vs. Meds/SA	4 (203/174)	MD:-0.11(−0.24, 0.01)	−1①	−1⑤	0	−1③	−1②	Very low
	Liu 2020 (32)	acu vs. Meds	3 (88/86)	SMD: −0.83 (−1.62, −0.05)	−1①	−1⑤	0	−1③	−1②	Very low
		acu + Meds vs. Meds; acu + CHM + Meds vs. CHM + Meds	3 (113/111)	SMD: −0.65 (−0.92, −0.38)	−1①	0	0	−1③	−1②	Very low
	Zheng 2022 (34)	acu + IVF-ET vs. IVF-ET; acu + FET vs. SA + FET; acu + FET vs. FET; acu + IVF-ET vs. SA + IVF-ET; acu + Meds vs. Meds	9 (185/188)	SMD: −0.86 (−1.23, −0.48)	−1①	−1⑤	0	−1③	−1②	Very low
PI	Zhong 2019 (29)	acu vs. press bean plus electromagnetic warm lamp	1 (30/30)	SMD: −7.12 (−8.53, −5.71)	−1①	0	0	−1③	−1④	Very low
		acu + Meds vs. Meds/SA	4 (203/174)	SMD: −1.37 (−2.59, −0.16)	−1①	−1⑤	0	−1③	−1②	Very low
	Liu 2020 (32)	acu vs. Meds	3 (88/86)	SMD: −1.08 (−1.74, −0.43)	−1①	−1⑤	0	−1③	−1②	Very low
		acu + Meds vs. Meds; acu + CHM + Meds vs. CHM + Meds	3 (113/111)	SMD: −0.68 (−0.95, −0.41)	−1①	0	0	−1③	−1②	Very low
	Zheng 2022 (34)	acu + IVF-ET vs. IVF-ET; acu + FET vs. SA + FET; acu + FET vs. FET; acu + Meds vs. Meds	10 (440/412)	SMD: −1.33 (−1.93, −0.72)	−1①	−1⑤	0	0	−1②	Very low
S/D	Zhong 2019 (29)	acu vs. Meds; acu + Meds vs. Meds	2 (103/84)	SMD: −0.60 (−0.89, −0.30)	−1①	0	0	−1③	−1④	Low
	Zheng 2022 (34)	acu + FET vs. SA + FET; acu + IVF-ET vs. IVF-ET; acu + Meds vs. Meds	4 (169/171)	SMD: −1.91 (−3.08, −0.75)	−1①	−1⑤	0	−1③	−1②	Very low

PR, pregnancy rate; CPR, clinical pregnancy rate; BPR, biochemical pregnancy rate; LBR, live birth rate; EMP, endometrial pattern; EMT, endometrial thickness; RI, resistive index; PI, endometrial pulse index; S/D, peak systolic velocity/ end-diastolic blood velocity; acu, acupuncture (including one of manual acupuncture, warm acupuncture, transcutaneous electrical acupuncture stimulation, plum-blossom needle treatment, electroacupuncture, cupping, auricular point sticking, auricular acupuncture, acupoint injection, acupoint catgut embedding, and moxibustion); Meds, medicine (including one of or some combinations of diane-35, metformin, placebo orally, human chorionic gonadotrophin, bromocriptine, progesterone, ethinylestradioland cyproterone acetate tablets, hormone replacement therapy, levofloxacin, dydrogesterone, letrozole, clomiphene, estradiol valerate tablets, controlled ovarian hyperstimulation, aspirin and fallopian tube injection of gentamicin, dexamethasone, chymotrypsin, sodium chlorideL); SA, sham acupuncture (including mock transcutaneous electrical acupuncture stimulation, sham electroacupuncture, placebo acupuncture, placebo auricular acupuncture, placebo moxibustion acupuncture); CHM, Chinese herbal medicine; IVF-ET, in vitro fertilization and embryo transfer; IST, intrauterine shortwave therapy; FET, frozen embryo transfer; RR, risk ratio; OR, odds ratio; SMD, standardized mean difference; MD, mean difference; ① The included studies have a large bias in methodology such as blinding and incomplete outcome data; ② Asymmetrical funnel plot suggests there may be a large publication bias; ③ The optimal information size is not enough; ④ Few studies are included and there may be a large publication bias; ⑤ The size and direction of the effect size and the overlap of the confidence interval are small, the p-value of the heterogeneity test is small, and the combined result of I^2^ value is large; ⑥ Large effect: RR>2; ⑦ This outcome index cannot directly represent the therapeutic effect of acupuncture on infertile women.

### 3.4 Efficacy and safety of acupuncture for infertile women

The efficacy of acupuncture on ER in infertile women is summarized in Table 5. Efficacy and safety were mainly reported based on the following outcomes: fertility, ER, and safety.

#### 3.4.1 Efficacy of acupuncture on fertility outcomes

##### 3.4.1.1 PR

There were seven SRs reported acupuncture on pregnancy rate, of which one SR (32) reported that there was no significant difference in improving pregnancy rate between acupuncture and medicine with very low-quality evidence, while acupuncture plus medicine alone or with CHM could improve the pregnancy rate with moderate-quality evidence. The other six SRs all indicated that acupuncture alone or with other treatments could improve the PR; of those, two had high-quality evidence (33, 37), three had low-quality evidence (29, 31, 36), and one had very low-quality evidence (34).

##### 3.4.1.2 CPR

One SR with very low-quality evidence reported that acupuncture plus COH could improve CPR, but there was no significant difference compared to COH therapy alone (38), while two SRs with low-quality evidence suggested that acupuncture as an adjuvant treatment resulted in a higher CPR than treatment without acupuncture treatment (30, 35).

##### 3.4.1.3 BPR

Two SRs suggested that acupuncture with medicine/FET had a significant effect on BPR compared to medicine/FET alone, sham acupuncture/blank control, or sham acupuncture plus medicine with low-quality evidence (30, 35).

##### 3.4.1.4 LBR

Two SRs with low-quality evidence and one SR with very low-quality evidence showed that compared with the control group, acupuncture alone or with other treatments (FET, IVF-ET) had no significant difference in LBR (29, 34, 35).

##### 3.4.1.5 Ovulation rate

One SR (37) with moderate-quality evidence and two SRs (31, 32) with low-quality evidence showed a statistically significant increase in the ovulation rate between the experimental and control groups, indicating that acupuncture can significantly increase the ovulation rate. One SR (36) reported that acupuncture plus CHM, with or without medicine, could not improve the ovulation rate compared to medicine alone with very low-quality evidence.

##### 3.4.1.6 Embryo transfer

Low-quality evidence (30) suggested that there was no statistically significant difference in the number of embryo transfers among the acupuncture treatment group, sham/placebo, or non-acupuncture group, while very low-quality evidence (29) suggested that compared with medication, acupuncture was effective in improving embryo transfer rates.

##### 3.4.1.7 Embryo implantation

Two SRs (30, 38) with low-quality evidence reported statistical significance in the embryo implantation rate, which indicated that acupuncture treatment was prior to the sham/placebo or non-acupuncture groups.

#### 3.4.2 Efficacy of acupuncture on ER outcomes

##### 3.4.2.1 EMP

Three SRs used type A endometrium as an evaluation indicator, whereas one SR used type A and type B endometrium as evaluation indicators. Four SRs (29, 30, 34, 35) reported that acupuncture could significantly improve the number of trilinear endometria when comparing acupuncture plus IVF-ET/FET/medicine with IVF-ET/FET/medicine with or without SA; of these, two had low-quality evidence and two had very low-quality evidence.

##### 3.4.2.2 EMT

Nine SRs reported acupuncture in EMT, of which one SR (29) showed that acupuncture alone was not statistically significant compared to medicine/SA/routine care with very low-quality evidence. However, when acupuncture was used as an add-on treatment combined with medicine, IVF-ET, FET, or CHM, there was a statistically significant difference between the experimental and control groups, indicating that with acupuncture, the EMT could be significantly improved, and the quality of evidence of SRs varied from low to very low (29–31, 33–38).

##### 3.4.2.3 RI

Three SRs (29, 32, 34) with very low-quality evidence found that acupuncture alone or in combination with medicine, IVF-ET, FET, or Chinese herbal medicine could decrease PI compared to the control group.

##### 3.4.2.4 PI

One SR (32) with very low-quality evidence, reported no significant difference when acupuncture was combined with other treatments (medicine and CHM). Two SRs (29, 32) with low-quality evidence suggested that, compared with press bean plus electromagnetic warm lamp and medicine, acupuncture showed a better improving effect on PI. Furthermore, very low-quality evidence (34) showed that acupuncture combined with IVF/TEF/medicine improved the PI better than a control without acupuncture.

##### 3.4.2.5 S/D

One SR (29) with low-quality evidence showed that, compared with medicine, acupuncture showed a better improving effect on S/D. Very low-quality evidence (34) showed that acupuncture combined with IVF-ET/FET/medicine was better than control without acupuncture.

#### 3.4.3 Safety outcomes

Of the 10 SRs, six SRs (29–31, 35, 37, 38) mentioned the adverse events of acupuncture in the treatment of infertile women, and one SR (32) showed no adverse effects during acupuncture therapy. The main adverse reactions included fainting, dizziness, and subcutaneous congestion at acupuncture points.

## 4 Discussion

Although all the included SRs revealed the potential benefits of acupuncture as an adjuvant treatment on fertility outcomes and ER outcomes in infertile women, the confidence in this finding is still reduced by the restrictions of the RCTs included in each SR. The AMSTAR2, ROBIS, and PRISMA-A assessments revealed significant qualitative deficiencies for each SR.

Evidence strength was categorized as moderate confidence when supported by high- or moderate-quality studies (40). This relatively reliable evidence suggests that combining acupuncture with other treatments (medicine, CHM, or IVF-ET) improves PR and ovulation rates compared to control groups not receiving acupuncture. However, conclusions are constrained by very low certainty evidence, and efficacy of acupuncture alone remains unproven. Therefore, the current evidence regarding acupuncture’s efficacy on ER outcomes is of insufficient confidence to draw meaningful conclusions.

AMSTAR 2 assessment revealed common methodological flaws, including: inadequate description of PICO elements; lack of a prior study protocol; insufficient justification for the SR type; non-comprehensive literature search strategy; absence of duplicate study selection/data extraction; incomplete listing of study exclusion rationales; insufficient description of included study characteristics; failure to report funding sources for included RCTs; inadequate discussion of risk of bias impact on overall findings; failure to identify or discuss sources of heterogeneity and their impact; and omission of conflict of interest reporting. The ROBIS assessment identified significant concerns, particularly in Phase 2 (Domains 2 and 4), indicating shortcomings in study identification, selection, data collection, and appraisal. PRISMA-A results highlighted three major reporting deficiencies: lack of protocol/registration reporting, inadequate description of information sources, and failure to account for funding sources’ roles. Finally, according to GRADE, the most prevalent reasons for downgrading evidence certainty were risk of bias, followed by publication bias, imprecision, and inconsistency.

Female infertility is closely associated with abnormal cervical mucus production, diminished oocyte quality, ovulatory dysfunction, tubal obstruction, and poor ER. ER plays a critical role in achieving successful pregnancy, with conception and pregnancy rates strongly correlated with endometrial thickness and blood flow patterns across different age groups (41). Acupuncture is widely used as an adjuvant therapy for infertility, particularly with treatments like IVF-ET. Our results indicate that adjuvant acupuncture may improve pregnancy rates and ovulation rates in infertile women. Clinical studies further demonstrate that acupuncture significantly improves clinical pregnancy rate, live birth rate, cycle ovulation rate, fertilization rate, oocyte yield, and high-quality embryo count, while reducing the incidence of ovarian hyperstimulation syndrome (42–52). Mechanistically, acupuncture appears to regulate female reproductive hormones, cellular functions, and immune signaling molecules, thereby supporting reproductive endocrine system regulation, follicular development, and embryo implantation (53). Specific studies on PER reveal that acupuncture enhances ER by improving endometrial morphology, promoting microcirculation, and bidirectionally modulating estrogen, progesterone, and their receptors (15, 18–22). However, owing to the limitations and inconsistencies of the current evidence, our overview shows that the efficacy of acupuncture on ER outcomes remains uncertain. *Zheng* conducted a SR and meta-analysis to evaluate dose-related acupuncture in infertile women with PER and showed that the effect of acupuncture was dose-dependent, and a trend of relatively higher acupuncture dosage showed better effects for PER (34). Historically, the efficacy of traditional Chinese acupuncture is closely related to the selection of acupoints, treatment duration, and treatment frequency (54), that is to say, dose-related acupuncture plays a critical role in the therapeutic effect of acupuncture. Therefore, future studies on the efficacy of acupuncture at different doses should be conducted. In addition, the SRs included in our overview revealed that acupuncture had better therapeutic effects when combined with other treatments, including medications, CHM, and IVF-ET. A network meta-analysis of the best acupuncture regimen for ER in infertile women may be beneficial for the selection of acupuncture regimens in future high-quality clinical research and clinical practice.

In view of these shortcomings, the following conditions should be adopted for future SRs: First, the PICO component should be made explicit in the research questions and inclusion criteria in an SR. Second, the study protocol of a SR is strongly recommended to be drafted and registered before the start of the SR, which can help to minimize the potential bias in the review process and reporting. Third, the search for primary studies should take a comprehensive approach whenever possible, with the search for gray literature being particularly important. Fourth, a list of excluded literature should be provided in the study selection process to reduce data omissions. Fifth, SRs need to fully document the essential characteristics of the included RCTs to help determine the extent to which the results of different studies can be combined and to inform the analysis of heterogeneity and the application of the results. Last but not least, SRs should report on their funding and the funding of the included RCTs to help identify potential conflicts of interest.

This comprehensive and systematic overview presented the most up-to-date evidence for the application of acupuncture for ER in infertile women, and registration on the PROSPERO platform was performed to limit the possibility of biased decision-making during this overview. However, some limitations exist in this study: (1) Language bias (Chinese/English-only inclusion); (2) Potential publication bias due to exclusion of gray literature; (3) Clinical heterogeneity precluded quantitative synthesis; (4) Inclusion of different acupuncture modalities-grouping together diverse interventions might bias synthesis.

## 5 Conclusion

Current evidence suggests that acupuncture, as an adjuvant therapy combined with other treatments (medication, IVF-ET, FET, or CHM), may benefit infertile patients and improve fertility outcomes. However, due to methodological limitations and inconsistent findings, definitive conclusions regarding acupuncture’s efficacy and safety for ER in infertile women cannot be drawn. Further high-quality research is required to substantiate acupuncture’s application for improving ER in this population. Future studies should: adopt core outcome sets (e.g., EMT, RI, live birth rate) to standardize ER assessment; implement harmonized acupuncture protocols or dosage standards; and prioritize RCTs designed to isolate acupuncture’s specific contribution from adjuvant effects (e.g., acupuncture vs. sham acupuncture + standardized IVF).

## Data Availability

The original contributions presented in the study are included in the article/Supplementary material, further inquiries can be directed to the corresponding author.

## Footnotes

^1^ http://www.guidelinedevelopment.org/
