# Supplementary material for: The efficacy of acupuncture on endometrial receptivity in infertile women: an overview of systematic review and meta-analysis
Source: Front Med (Lausanne). 2025 Aug 28;12:1609519. doi: 10.3389/fmed.2025.1609519 (PMC12424591; doi:10.3389/fmed.2025.1609519)
Supplement: Supplementary file 1 [file Data_Sheet_1.docx]

## **Supplementary 1.** Search strategies of 4 English databases

## **（1）PubMed search strategy (March 1, 2025)**

#1 ((((((((((((((((((((infertility) OR (fertility disorder)) OR (infecundity)) OR (primary infertility)) OR (secondary infertility)) OR (sexual sterility)) OR (sexual sterility)) OR (subfertility)) OR (hypofertility)) OR (reduced fertility)) OR (sub-fertility)) OR (in vitro fertilization)) OR (test-tube fertilization)) OR (test tube fertilization)) OR (test-tube babies)) OR (test tube babies)) OR (intracytoplasmic sperm injection)) OR (icsi)) OR (artificial insemination)) OR (ovulation induction)) OR (assisted reproduction)

#2 (((((((((((((((endometrium) OR (endometrial epithelium)) OR (endometrium epithelium)) OR (endometrium extract)) OR (tunica mucosa uteri)) OR (uterine endometrium)) OR (uterine mucosa)) OR (uterus endometrium)) OR (uterus mucosa)) OR (endometria)) OR (endometrial receptivity)) OR (endometria receptivity)) OR (uterine artery)) OR (uterine arteries)) OR (endometrium blood flow)) OR (pulse index or resistive index)

#3 ((((((((((((Acupuncture Therapy) OR (Acupuncture Treatment)) OR (Acupuncture Treatments)) OR (Pharmacoacupuncture Treatment)) OR (Pharmacoacupuncture Therapy)) OR (Acupotomy)) OR (Acupotomies)) OR (Acupuncture)) OR (Pharmacopuncture)) OR (acupuncture point)) OR (acupressure)) OR (electroacupuncture)) OR (moxibustion)

#4 (systematic review) OR (meta analysis)

#5 #1 AND #2 AND #3 AND #4

**（2）EMBASE search strategy search strategy (March 1, 2025)**

#1 'infertility'/exp OR infertility OR 'fertility disorder'/exp OR 'fertility disorder' OR 'infecundity'/exp OR infecundity OR 'primary infertility'/exp OR 'primary infertility' OR 'secondary infertility'/exp OR 'secondary infertility' OR 'sexual sterility'/exp OR 'sexual sterility' OR 'reproductive sterility' OR 'subfertility'/exp OR subfertility OR 'hypofertility'/exp OR hypofertility OR 'reduced fertility'/exp OR 'reduced fertility' OR 'sub fertility'/exp OR 'sub fertility' OR 'in vitro fertilization'/exp OR 'in vitro fertilization' OR 'test-tube fertilization' OR 'test tube fertilization' OR 'test-tube babies' OR 'test tube babies' OR 'intracytoplasmic sperm injection'/exp OR 'intracytoplasmic sperm injection' OR 'icsi'/exp OR icsi OR 'artificial insemination'/exp OR 'artificial insemination' OR 'ovulation induction'/exp OR 'ovulation induction' OR 'assisted reproduction'/exp OR 'assisted reproduction'

#2 'acupuncture therapy'/exp OR 'acupuncture therapy' OR 'acupuncture treatment' OR 'acupuncture treatments' OR 'pharmacoacupuncture treatment' OR 'pharmacoacupuncture therapy' OR 'acupotomy'/exp OR acupotomy OR acupotomies OR 'acupuncture'/exp OR acupuncture OR 'pharmacopuncture'/exp OR pharmacopuncture OR 'acupuncture point'/exp OR 'acupuncture point' OR 'acupressure'/exp OR acupressure OR 'electroacupuncture'/exp OR electroacupuncture OR 'moxibustion'/exp OR moxibustion

#3 'endometrium'/exp OR endometrium OR 'endometrial epithelium'/exp OR 'endometrial epithelium' OR 'endometrium epithelium'/exp OR 'endometrium epithelium' OR 'endometrium extract'/exp OR 'endometrium extract' OR 'tunica mucosa uteri'/exp OR 'tunica mucosa uteri' OR 'uterine endometrium'/exp OR 'uterine endometrium' OR 'uterine mucosa'/exp OR 'uterine mucosa' OR 'uterus endometrium'/exp OR 'uterus endometrium' OR 'uterus mucosa'/exp OR 'uterus mucosa' OR endometria OR 'endometrial receptivity'/exp OR 'endometrial receptivity' OR 'endometria receptivity' OR 'uterine artery'/exp OR 'uterine artery' OR 'uterine arteries' OR 'endometrium blood flow'/exp OR 'endometrium blood flow' OR 'pulse index'/exp OR 'pulse index' OR 'resistive index'/exp OR 'resistive index'

#4 'systematic review'/exp OR 'systematic review' OR 'meta analysis'/exp OR 'meta analysis'

#5 1 and 2 and 3 and 4

**（3）Web of Science** **search strategy (March 1, 2025)**

#1TS=(infertility OR Sterility, Reproductive OR Sterility Reproductive OR Sterility OR Subfertility OR Sub-Fertility OR infertil* OR subfertil* OR ‘Fertilization in Vitro’ OR ‘In Vitro Fertilization’ OR ‘In Vitro fertilization’ OR Test-Tube OR Fertilization OR Fertilization, Test-Tube OR fertilization, Test-Tube OR Test Tube OR Fertilization Test-Tube OR fertilization OR fertilization in Vitro OR Test-Tube Babies OR Babies OR Test-Tube Baby OR Test-Tube OR Test Tube Babies OR Test-Tube Baby OR Sperm Injections, Intracytoplasmic OR Injection, Intracytoplasmic Sperm Injections OR Intracytoplasmic Sperm OR Intracytoplasmic Sperm Injection OR Sperm Injection, Intracytoplasmic OR Intracytoplasmic Sperm Injections OR ICSI OR Injections, Sperm, Intracytoplasmic OR intracytoplasmic sperm injection* OR artificial insemination* OR assisted reproduct* OR ovulation induc* OR infertil* OR assisted reproduct* OR ovari* adh2 stimulat*) and Preprint Citation Index (Exclude - Database)

#2 TS=(Acupuncture Therapy OR Acupuncture Treatment OR Acupuncture Treatments OR Pharmacoacupuncture Treatment OR Pharmacoacupuncture Therapy OR Acupotomy OR Acupotomies OR Acupuncture OR Pharmacopuncture OR acupuncture point OR acupressure OR electroacupuncture OR moxibustion) and Preprint Citation Index (Exclude - Database)

#3 TS=(Endometrium OR endometrial OR endometrium receptivity OR endometrial receptivity OR uterus OR Uteri OR Womb OR Wombs OR Uterus Cornua OR Uterine Cornua OR Cornua, Uterine OR Uterine Fundus OR Fundus, Uterine OR Fundus Uteri OR Fundus Uterus OR Uteri, Fundus OR uterine arteries OR Arteries, Uterine OR Artery, Uterine OR Uterine Arteries OR endometrium blood flow OR pulse index OR resistive index OR endometri* OR uter*) and Preprint Citation Index (Exclude - Database)

#4 TS=(systematic review OR meta analysis) and Preprint Citation Index (Exclude - Database)

#5 #4 AND #3 AND #2 AND #1 and Preprint Citation Index (Exclude - Database)

**（4）Cocharne search strategy (March 1, 2025)**

#1 MeSH descriptor: [Infertility] explode all trees

#2(infertilitySterility, Reproductive):ti,ab,kw OR (Sterility):ti,ab,kw OR (Reproductive Sterility):ti,ab,kw OR (Subfertility):ti,ab,kw

#3(vitro fertilization):ti,ab,kw OR (intrauterine insemination*):ti,ab,kw OR (artificial insemination*):ti,ab,kw OR (subfertil*):ti,ab,kw OR (assisted reproduct*):ti,ab,kw

#4 MeSH descriptor: [Acupuncture] explode all trees

#5 MeSH descriptor: [Acupuncture Points] explode all trees

#6 MeSH descriptor: [Acupuncture Therapy] explode all trees

#7 (Pharmacopuncture):ti,ab,kw OR (Pharmacoacupuncture Treatment):ti,ab,kw OR (Pharmacoacupuncture Therapy):ti,ab,kw OR (Acupotomy):ti,ab,kw OR (Acupotomies):ti,ab,kw

#8 (Acupoints):ti,ab,kw OR (Acupoint):ti,ab,kw OR (electroacupuncture):ti,ab,kw OR (moxibustion):ti,ab,kw OR (acupressure):ti,ab,kw

#9 MeSH descriptor: [Fertilization in Vitro] explode all trees

#10 (Baby, Test-Tube):ti,ab,kw OR (Test Tube Babies):ti,ab,kw OR (Test-Tube Baby):ti,ab,kw OR (Injection, Intracytoplasmic Sperm):ti,ab,kw OR (Injections, Intracytoplasmic Sperm):ti,ab,kw

#11 (Intracytoplasmic Sperm Injection):ti,ab,kw OR (Sperm Injection, Intracytoplasmic):ti,ab,kw OR (Intracytoplasmic Sperm Injections):ti,ab,kw OR (ICSI):ti,ab,kw OR (Injections, Sperm, Intracytoplasmic):ti,ab,kw

#12 MeSH descriptor: [Sperm Injections, Intracytoplasmic] explode all trees

#13 (In Vitro Fertilization):ti,ab,kw OR (In Vitro Fertilizations):ti,ab,kw OR (Test-Tube Fertilization):ti,ab,kw OR (Fertilization, Test-Tube):ti,ab,kw OR (Fertilizations, Test-Tube):ti,ab,kw

#14 (Test Tube Fertilization):ti,ab,kw OR (Test-Tube Fertilizations):ti,ab,kw OR (Fertilizations in Vitro):ti,ab,kw

#15 #1 OR #2 OR #3 OR #9 OR #10 OR #11 OR #12 OR #13 OR #14

#16 #4 OR #5 OR #6 OR #7 OR #8

#17 MeSH descriptor: [Endometrium] explode all trees

#18 MeSH descriptor: [Uterine Artery] explode all trees

#19 (endometria):ti,ab,kw OR (endometrial):ti,ab,kw OR (endometrial receptivity):ti,ab,kw OR (endometria rceptivity):ti,ab,kw OR (Uteri):ti,ab,kw (Word variations have been searched)

#20 (Uterine Arteries):ti,ab,kw OR (endometrium blood flow):ti,ab,kw OR (thin endometrium):ti,ab,kw OR (Arteries, Uterine):ti,ab,kw OR (Artery, Uterine):ti,ab,kw

#21 (endometrium thickness):ti,ab,kw OR (endometrium pattern):ti,ab,kw OR (endometrium contraction):ti,ab,kw OR (Arteries, Uterine):ti,ab,kw OR (pulse index):ti,ab,kw

#22 (pulse index):ti,ab,kw OR (resistive index):ti,ab,kw OR ("uterine artery"):ti,ab,kw OR ("uterine blood flow"):ti,ab,kw OR (endometri*):ti,ab,kw

#23 MeSH descriptor: [Systematic Review] explode all trees

#24 ("systematic review"):ti,ab,kw OR ("meta-analyses"):ti,ab,kw

#25 #17 OR #18 OR #19 OR #20 OR #21 OR #22

#26 #23 OR #24

#27 #15 AND #16 AND #25 AND #26

## **Table S1.** List of excluded studies.

| Author/Year | Title | Reason for exclusion |
| --- | --- | --- |
| Gu et al., 2019 | *The effects of acupuncture on pregnancy outcomes of in vitro fertilization with embryo transfer: An interdisciplinary systematic review* | SR with no outcome related to endometrial receptivity |
| Yang et al., 2023 | *Meta analysis of ovulation induction effect and pregnancy outcome of acupuncture & moxibustion combined with clomiphene in patients with polycystic ovary syndrome* | SR related to network meta-analysis |
| Hu et al., 2021 | *Complementary and Alternative Medicine for the Treatment of Abnormal Endometrial Conditions in Women with PCOS: A Systematic Review and Meta-Analysis of Randomized Controlled Trials* | SR included only one RCT related to acupuncture for infertile women |
